# UHPLC Q-Exactive MS-Based Serum Metabolomics to Explore the Effect Mechanisms of Immunological Activity of *Astragalus* Polysaccharides With Different Molecular Weights

**DOI:** 10.3389/fphar.2020.595692

**Published:** 2020-12-15

**Authors:** Ke Li, Lian-Jie Cui, Yu-Xin Cao, Shu-Ying Li, Li-Xia Shi, Xue-Mei Qin, Yu-Guang Du

**Affiliations:** ^1^ Modern Research Center for Traditional Chinese Medicine, Shanxi University, Taiyuan, China; ^2^ Institute of Process Engineering, Chinese Academy of Sciences, Beijing, China; ^3^ College of Chemistry and Chemical Engineering, Shanxi University, Taiyuan, China

**Keywords:** *Astragalus* polysaccharides, structural analysis, metabolomics, serum, immunomodulation, differential metabolites

## Abstract

*Astragalus* polysaccharides (APS) have a wide range of biological activities. Most researchers discuss total APS as the main research object. However, because the relative molecular weight of APS has a wide distribution, in-depth studies on the mechanisms of the biological activity of notable molecules are limited. For example, the relationship between the immunomodulatory effect of APS and its relative molecular weight has not been clearly defined. Therefore, in this paper, we separated and obtained APS of different molecular weights by ultrafiltration technology and then constructed a mouse cyclophosphamide-induced immunosuppression model to investigate the immune activity of APS of different molecular weights. The immune enhancement mechanism of APS was explored by examining changes in routine blood indicators, body weight, immune organs, and differential metabolites in mouse serum. Results showed that APS-I (molecular weight, >2,000 kDa), APS-II (molecular weight, 1.02 × 10^4^ Da) and APS-III (molecular weight, 286 Da) could increase the number of immune cells in mouse serum and improve immune organ damage to varying degrees. Among the samples obtained, APS-II showed the best effects. Compared with those in the blank group, 29 metabolites determined by UHPLC Q-Exactive MS in the serum of the model group changed remarkably, and APS-I, APS-II, and APS-III respectively restored 13, 25, and 19 of these metabolites to normal levels. Metabolomics analysis revealed that APS-II is mainly responsible for the immunomodulatory activity of APS. Metabolomics analysis revealed that the mechanisms of this specific molecule may involve the regulation of phenylalanine metabolism, cysteine and methionine metabolism, tricarboxylic acid cycle (TCA cycle) and arginine and proline metabolism.

## Introduction

*Astragali Radix* (AR) refers to the dried root of the perennial legume *Astragalus mongholicus* Bunge, which is a traditional Chinese medicine. It is listed as an upper-grade material in Shen Nong’s Herbal Classic and has been used as an immunomodulator in traditional Chinese medicine prescriptions for over 2,000 years. AR has a wide range of uses; for example, it can invigorate the qi, solidify surfaces, reduce water retention and swelling, and support sore muscles. The Ministry of Commerce of China has listed AR as among the country’s 60 strategic key Chinese medicinal materials and 18 main Chinese medicinal materials. In addition, in 2018, AR was included in the Chinese medicine and food homology list.

Modern phytochemical and pharmacological experiments have shown that polysaccharides have very strong immunomodulatory activity and are highly abundant in AR (Wu X. et al., 2011). Complex polysaccharide mixtures may be obtained by water extraction and alcohol precipitation from AR. *Astragalus* polysaccharides (APS) are composed of polysaccharides with diverse structures and different molecular weights distributed over a wide range (5.6 × 10^3^–4.8 × 10^6^ Da) (Jin et al., 2014). The molecules of APS are mainly composed of different types of functional groups, including dextrans and arabinogalactans.

Research on the activity of APS often describes their ability to regulate non-specific immunomodulation, enhancement of specific and non-specific immunity. APS are known to have a variety of biological activities, such as antitumor, antidiabetic, antiviral, cardiovascular, and neuroprotective effects. Studies have shown that these pharmacological activities generally involve immunomodulation (Morten et al., 2008; Jiang et al., 2013; Li et al., 2017). Unfortunately, most available studies focus on total APS or a certain component of APS (Table 1). At present, reports on the separation of total APS according to molecular weight and the structural analysis and immunological screening of the resulting components are scarce. Therefore, clarifying the relationship between the molecular weight, structure and activity of different APS components is necessary to elucidate the medicinal material basis of AR.

**TABLE 1:** Structure characterization of *Astragalus* polysaccharides (APS).

No.	Name	Molecular weight	Main structure	Bioactivity	References
1	LMw-APS	5.6 × 10^3^	Backbone composed of (1→4) linked glucose and some (1→2) linked Xyl and (1→3) linked GalA	Immunomodulatory activity	Qu (2010)
2	APS	2 × 10^4^–6 × 10^4^	(1→4)-α-glucan, arabinogalactan, rhamnose-galacturonic acid glycan, and arabinose-galactose proteoglycan	Immunomodulatory activity	Qing-yang et al. (2017)
3	APS	2.07 × 10^4^	(1→4)-linked Glc backbone with a (1→6)-linked Glc branch	Antioxidant and immunomodulation	Niu et al. (2011)
4	APS	8.7 × 10^3^–4.8 × 10^6^	Backbone composed of α-(1→3) glucose and some (1→4), (1→6) glucoses	Antitumor	Zhen-yuan et al. (2011)
5	AMon-S	7.6 × 10^4^	Mainly α-arabino-β-3,6-galactan-type structural units	Reticuloendothelial system-potentiating activity	Shimizu et al. (1991)
6	APS	1.6 × 10^6^	Glucose, galactose, mannose, and arabinose	Antioxidant activity	Yan et al. (2010)
7	APS	1.2 × 10^4^–3.6 × 10^4^	Backbone mainly composed of α-d-(1→4)-linked Glc	Antiatherosclerosis and antidiabetes	Wang et al. (2010)
8	cAMPs-1A	1.23 × 10^4^	α-glycosidic linkage	Immunomodulation and antitumor	An-Jun et al. (2017)
9	A2Nb	6.99 × 10^4^–1.58 × 10^5^	Arabinogalactan	Immunomodulation	Wu X. et al. (2011)
10	APS4	1.23 × 10^4^	(1→2)-α-d-Glcp and (1→2,6)-α-d-Glcp	Antitumor	Yu et al. (2019)

To clarify differences in the ability of APS components of different molecular weights and structures to regulate the immune activity of total APS, we characterized total APS according to its molecular weight distribution by gel chromatography and then used ultrafiltration to prepare APS of different molecular weights. A mouse cyclophosphamide-induced immunosuppression model was used to screen differences in the activity of each component obtained to determine the most immunologically active samples. The serum metabolomic strategy based on liquid chromatography (LC)–high-resolution mass spectrometry (MS) was used to study the effects of APS of different molecular weights on the immune capacity of immunosuppressed mice and analyzed the metabolic regulation pathways involved to clarify their regulatory mechanisms. Compared with ordinary liquid MS systems, the Thermo Scientific Q Exactive bench-top LC–MS/MS instrument used in this research combines, with a resolution of up to 140,000. The instrument can determine the masses of various metabolites with high accuracy, identify the relevant molecular formulas and structural characteristics of these metabolites, and provide a reliable qualitative and quantitative approach to detect compounds. This equipment is a powerful tool in the serum metabolomics study of APS immunomodulation (Rousu et al., 2010; Li et al., 2020; Prasad et al., 2011; Miggiels et al., 2018).

## Materials and Methods

### Materials

IKA RH digital magnetic stirrer was purchased from IKA (Germany); Laboratory miniature nanofiltration system GS−NF500 was purchased from Shanghai Guxin Biological Technology Co., Ltd (Shanghai, China); Huapu S6000 high-performance liquid chromatograph and chromachem evaporative light scattering detector were purchased from Acchrom Tech (Beijing, China); Thermo Finnigan Trace GC + Polaris Q gas chromatography-thermo finnigan, high-resolution liquid mass spectrometer (Thermo Scientific Q Exactive), Xcalibur software, acetonitrile (mass spectrometry grade) and formic acid (mass spectrometry grade) were purchased from Thermo-Fisher Scientific (USA); Bruker Tensor 27 infrared spectrometer was purchased from Bruker (Germany); FD-1D-80 vacuum freeze dryer was purchased from Xian Depai Biotechnology Co., Ltd. (Xian, China); Sartorius BSA124S analytical balance was purchased from Sartorius (Germany); TGL-16 high-speed tabletop refrigerated centrifuge was purchased from Hunan Xiangyi Centrifuge Instrument Co., Ltd (Hunan, China); dextran standard (Mw: 180, 2,700, 5,250, 9,750, 13,050, 36,800, 64,650, 135,350, 300,600, 2,000,000 Da) was purchased from China National Institute of Pharmaceutical and Biological Products (Beijing, China); glucose (Glc), galactose (Gal), arabinose (Ara), rhamnose (Rha), mannose (Man), fucose (Fuc), galacturonic acid (GalA), *N*-acetylglucosamine (GlcNAc) were purchased from Meilun Biotechnology (Dalian, China); papain (Solarbio), trichloroacetic acid, trifluoroacetic acid (TFA), anhydrous dimethyl sulfoxide (DMSO) were purchased from Aladdin (Shanghai, China); 1-phenyl-3-methyl-5-pyrazolone (PMP), deuterated sodium borohydride (NaBD_4_) were purchased from Sigma (USA).

Shanxi Hunyuan AR (the dried root of *Astragalus mongholicus* Bunge) was harvested in 2017 after 5 years of growth and identified by Prof. Xue-mei Qin of Shanxi University. Samples of the medicinal materials used in this work were kept in the sample library of the Modern Research Center of Traditional Chinese Medicine of Shanxi University.

### Separation and Preparation of Total APS

The AR powder was passed through a 100 mesh sieve. Deionized water was added to 15 g of AR powder at a ratio of 1:20, and the mixture was stirred with a magnetic stirrer at 90°C for 4 h for extraction. After centrifugation, the supernatant was concentrated to 150 ml and subjected to enzymolysis with trichloroacetic acid to remove proteins. Here, 200 U of papain was added to concentrate, and the mixture was placed in a water bath at 45°C for 6 h. Next, 50 ml 10% trichloroacetic acid was added to concentrate, and the mixture was stirred in an ice bath for 15 min and allowed to stand for 30 min. Centrifugation was then carried out to separate the precipitate. After centrifugation, absolute ethanol was slowly added to the supernatant to achieve a final alcohol concentration of 90%. The 90% ethanol solution was allowed to stand overnight, and centrifuged to obtain the precipitate, the precipitate was freeze-dried to obtain APS, and the purity and molecular weight of this product were calculated.

### Determination of the Polysaccharide Content of Total APS

The phenol–sulfuric acid method was used to determine the content of polysaccharides in APS.

Standard curve drawing: Exactly 0, 0.1, 0.2, 0.4, 0.6, 0.8, and 1.0 ml of a 100 μg ml^−1^ Glc reference solution were accurately measured, respectively placed in test tubes with stopper, and added with water to 1 ml. Then, 1 ml of 5% phenol solution and 5 ml of concentrated sulfuric acid were added to the tubes. The solutions were mixed by vortexing, boiled in a water bath for 15 min, immersed in an ice bath, and then allowed to reach room temperature. The absorbance (A) of the solutions was measured at 490 nm. This experiment was repeated thrice. A was considered the ordinate and the Glc concentration (C) was considered the abscissa to obtain the linear regression equation of Glc.

Determination of sample polysaccharide content: Each group of polysaccharide samples was prepared as a test solution of 100 μg ml^−1^. Exactly 1.0 ml of each solution was obtained and treated in the same manner as the standard products.

### Determination of the Protein Content of Total APS

The Coomassie brilliant blue method was used to determine the protein content of total APS.

Standard curve drawing: A 500 μg ml^−1^ standard protein solution was prepared. Aliquots of 0, 5, 10, 20, 40, 60, 80, and 100 μl of this protein reference solution were placed in EP tubes, added with distilled water to 100 μl, added with 1 ml of G250 staining solution, shaken well, and allowed to stand for 5 min. The absorbance of each solution was measured at 595 nm. The absorbance (A) was taken as the ordinate and the standard protein solution concentration (C) was considered the abscissa to obtain a linear regression equation.

Determination of sample protein content: The polysaccharide sample was accurately weighed to prepare a 500 μg ml^−1^ test solution. Exactly 100 μl of this solution was obtained and treated in the same manner as the standard products.

### Determination of Molecular Weight Distribution of Total APS

The relative molecular mass of APS was determined by high-performance gel filtration chromatography (HPGFC). Chromatographic conditions: column, TSK-GMPWXL (10 μm, 7.8 mm × 300 mm) gel column (Tosoh Corporation, Japan); drift tube temperature, 90°C; atomizer (N_2_) pressure, 26 psi; mobile phase, water; flow rate, 0.5 ml min^−1^; column temperature, 30°C; injection volume, 20 μl.

Standard curve drawing: Ten dextran reference substances with known relative molecular masses were diluted with the mobile phase to prepare standard solution of 5 mg ml^−1^ as the reference solution. The retention time (*t*_R_) was taken as the abscissa, lgMw was considered the ordinate, and the linear regression equation of the dextran reference substance was obtained.

Determination of molecular weight of polysaccharide sample: The polysaccharide sample was diluted with water to prepare a 5 mg ml^−1^ test solution. The retention time of the polysaccharide sample was substituted into the regression equation of the standard product to obtain the molecular weight distribution of total APS.

### Separation and Preparation of APS Components of Different Molecular Weights

The molecular weight distribution chromatogram of total APS indicated that the molecular weight distribution of the compound could be mainly divided into three parts, namely, 2000 kDa (beyond the linear range), 10 kDa, and approximately 300 Da. APS was then formulated into a 5 mg mL^−1^ solution and divided into APS-I (molecular weight, >30 kDa), APS-II (molecular weight, 1–30 kDa), and APS-III (molecular weight, <1 kDa) by using ultrafiltration membranes with molecular weight cutoffs of 30 and 1 kDa. Each component was concentrated and freeze-dried.

### Determination of the Monosaccharide Composition of APS of Different Molecular Weights

Exactly 5 mg of APS of different molecular weights was weighed, the established method in our laboratory was used to determine the monosaccharide composition (Cao et al., 2019; Cao et al., 2020). Experimental conditions: column, Huapu Unitary C18 columns (250 mm × 4.6 mm, 5 μm); mobile phase A, 50 mM potassium dihydrogen phosphate buffer (pH 6.7); mobile phase B, acetonitrile; flow rate, 1.0 ml min^−1^; column temperature, 35°C; detection wavelength, 250 nm; injection volume, 20 μl.

The polysaccharides were hydrolyzed as follows: Exactly 5 mg of the sample to be tested was weighed out, placed in a hydrolysis tube, added with 3 ml of 2 M trifluoroacetic acid for dissolution, sealed, and placed in a 120°C oven for 2 h for hydrolysis. The sample was taken out of the oven, cooled to room temperature, and then transferred to a round-bottom flask. A small amount of methanol was added repeatedly to the sample under reduced pressure to remove residual trichloroacetic acid.

Derivatization of the monosaccharide reference substance: Exactly 0.09, 0.106, 0.099, 0.09, 0.075, 0.082, 0.111, and 0.091 g of Man, GalA, Glc, Gal, Ara, Fuc, GlcNAc, and Rha reference substances were accurately weighed and placed into the same 10 ml centrifuge tube. Exactly 5 ml of ultrapure water was added to the tube to dissolve the solids and obtain a mixed solution of 100 mM. Exactly 1 ml of this mixed standard was added with 9 ml of ultrapure water to dilute the mixed standard to 10 mM. This solution was placed in a 20 ml measuring flask, and water was added to the mark to obtain the mixed reference solution. Exactly 0.2 ml of the mixed standard was added to a 2 ml EP tube, mixed with 0.24 ml of 0.5 M PMP and 0.2 ml of 0.3 M NaOH solution, shaken thoroughly, placed in a constant-temperature metal bath, and reacted at 70°C for 70 min at 300 rpm. The mixture was cooled to room temperature, added with 0.2 ml of 0.3 M HCl for neutralization, added 1 ml of chloroform for extraction, and then centrifuged. The organic layer was discarded, and extraction was repeated thrice. The upper aqueous solution was collected, filtered through a 0.45 μm microporous filter membrane, and then set aside.

Polysaccharide samples were derivatized as follows: Exactly 0.2 ml of the polysaccharide hydrolysate was obtained and processed according to the method used to derivatize the monosaccharide reference substance.

### Infrared Spectroscopy of APS of Different Molecular Weights

Exactly 2 mg of APS of different molecular weights was weighed, mixed with 100 mg of KBr powder, and compressed into tablets. The scanning wavelength range was 4,000–450 cm^−1^.

### Methylation Analysis of APS of Different Molecular Weights

Preparation of NaH-DMSO suspension: Exactly 5 mg of each polysaccharide sample was sufficiently dried and dissolved in anhydrous dimethyl sulfoxide (DMSO). The hydrolysis tube was filled with N_2_, and magnetic stirring was carried out overnight until the polysaccharide sample was completely dissolved. Next, 30 mg of dried NaH was quickly added to 3 ml of anhydrous DMSO. The hydrolysis tube was filled with N_2_, and magnetically stirred for 1 h to produce a uniform suspension.

Methylation: The NaH–DMSO suspension was added to the dissolved polysaccharide sample, sealed with N_2_, ultrasonically reacted for 1 h, and then reacted with 1 ml of iodomethane in the dark for 1 h. This reaction was repeated thrice and terminated by addition of 5 ml of water, and then extracted four times with 5 ml of CHCl_3_. The upper layer was discarded, and the bottom layer was extracted four times with 3 ml of water. The upper layer was discarded once more. Anhydrous Na_2_SO_4_ was added, filter and take the filtrate, and dried with N_2_. Take a small amount for infrared spectroscopy. The absence of an absorption peak at 3,400 cm^−1^ indicates the absence of –OH groups, which means methylation is complete. Otherwise, the reaction solution was dried and the methylation was repeated.

Hydrolysis: After methylation, added 2 ml of 2 mol l^−1^ trifluoroacetic acid into the methylated solution to react at 120°C for 90 min, and dried with N_2_. Then added 10 ml of methanol, and dried with N_2_, and repeated thrice.

Reduction reaction: After acid hydrolysis, 2.5 ml of 2% NaBD_4_ was added to the product, and the mixture was reacted on a shaker at 40°C for 90 min. Glacial acetic acid was slowly add to the mixture until bubbles ceased to form. Then, the mixture was air dried.

Acetylation: After reduction, 500 μl of acetic anhydride and 100 μl of 1-methylimidazole were added to the product, reacted at room temperature for 15 min, and then terminated by addition of 1.5 ml of water. After cooling, the product was extracted thrice with 0.5 ml of CH_2_Cl_2_. The lower layers were combined and extract with 1 ml of water. The upper layer was removed, anhydrous Na_2_SO_4_ was added into the lower layers, the mixture was filtered and dried, and then redissolved the product in 20 μl of CH_2_Cl_2_, and analyzed the product by GC–MS.

GC–MS analysis conditions: gas chromatography column, DB-5MS capillary column; inlet temperature, 220°C; carrier gas, helium; carrier gas flow rate, 1.0 ml min^−1^; split flow, 10:1.

Temperature program: starting temperature, 100°C; heating program, increased to 180°C at 5°C min^−1^ and held for 1 min, increased to 190°C at 1°C min^−1^ and held for 2 min, increased to 220°C at 30°C min^−1^ and held for 2 min, increased to 230°C at 1°C min^−1^ and held for 2 min, increased to 280°C at 20°C min^−1^ and held for 10 min. MS conditions (Prozil et al., 2012): ionization source, electron bombardment source; ion source temperature, 220°C; scan mode, full scan; scan range, 30–550 (m/z).

### Grouping and Treatment of Mice

Male BALB/c mice (SPF grade) aged 6–8 weeks and weighing 20 ± 2 g were purchased from Beijing Weitong Lihua Experimental Animal Technology Co., Ltd. (Animal License No. SCXK (Beijing) 2016-0006). The rearing environment had a temperature of 25 ± 2°C, humidity of 50% ± 10%, and 12 h/12 h alternating light/and dark illumination. The animal experiments conformed to the relevant regulations of the Shanxi University Scientific Research Ethics Review Committee.

The cyclophosphamide-induced immunosuppression mouse model was constructed by dividing the mice into groups (9 per group) into different groups as follows: control (C), model (M), positive drug (APS), APS-I, APS-II, and APS-III. During drug administration, the dosage of APS-I was 80 mg/kg/d, the dosage of APS-II was 100 mg/kg/d, the dosage of APS-Ⅲ was 20 mg/kg/d, the dosage of APS was 200 mg/kg/d, the dosage of polysaccharides of different molecular weights was converted into the equivalent of 200 mg/kg/d of total APS according to the mass percentage of the component in total APS (APS-I:APS-II:APS-Ⅲ = 40:50:10%). The C group was given the same amount of physiological water, administered intragastrically once a day for 14 days. On the ninth day of administration, all groups except the C group were given an intraperitoneal injection of 75 mg/kg cyclophosphamide (∼1.5 mg/body, 0.2 ml/body) for three consecutive days to obtain the immunosuppression model. The mice were intragastrically administered with different APS components until the 21st day. On the day after the last administration, the mice were sacrificed and their organs and serum were collected and stored for future use.

### Pharmacodynamic Evaluation

#### Effects of APS of Different Molecular Weights on the Blood Routine of Immunosuppressed Mice

Exactly 200 μL of blood was taken from each orbit of each mouse and analyzed by a blood cell analyzer for white blood cells, lymphocytes, red blood cells, hemoglobin, and platelets.

#### Effect of APS of Different Molecular Weights on the Body Weight of Cyclophosphamide Immunosuppressed Mice

The body weight of each mouse was recorded every other day, and the changes in body weight are analyzed.

#### Effect of APS of Different Molecular Weights on the Weight of Immune Organs in Cyclophosphamide Immunosuppressed Mice

The mice were sacrificed 24 h after the last drug administration, and the spleen and thymus indices of mice in the C, M, and drug administration groups were compared.

### Preparation of LC–MS Serum Samples

The serum samples were thawed at 4°C. Then, 100 μL of serum and 200 μL of 0.1% formic acid acetonitrile were vortexed in a 1.5 ml EP tube for 2 min and then centrifuged at 4°C and 13,000 rpm for 15 min to remove the protein. The supernatant was obtained for LC−MS analysis. Exactly 10 μL of each of the six groups of serum samples were mixed, and QC samples were prepared according to the above method.

### LC–MS Liquid Phase Conditions

Chromatography system, Dionex UltiMate 3000 UHPLC; chromatographic column, Waters Acquity UPLC HSS T3 column (2.1 mm × 100 mm, 1.7 µm); mobile phase A, 0.1% formic acid water; mobile phase B, 0.1% formic acid acetonitrile; flow rate: 0.2 ml min^−1^; injection volume: 5 μL; column temperature: 40°C. The mobile phase gradient was as follows: 0–2 min, 2% B; 2–3 min, 2–35% B; 3–17 min, 35–70% B; 17–18 min, 70% B; 18–29 min, 70–98% B; 29–31 min, 98% B; 31–32 min, 98−2% B, 32–35 min, 2% B.

### LC-MS Mass Spectrometry Conditions

Refer to the method of existing research (Du et al., 2019), mass spectrometry system, Q-Exactive/Focus; ionization method, ESI electrospray ionization; acquisition mode, positive and negative ion switching; scan mode, full scan/dd-MS^2^; m/z acquisition range, 100–1,500; positive electrode spray voltage, 3.5 kV; negative electrode spray voltage, 2.5 kV; capillary temperature, 320°C; heater temperature, 300°C; sheath gas flow rate, 35 arb; auxiliary gas flow rate, 10 arb; resolution setting, MS full scan 35,000 FWHM and MS/MS 17500 FWHM; NCE settings, 12.5, 25, 37.5 eV.

### Metabolite Identification and Data Processing

The collected LC–MS/MS raw data files were imported into Compound Discoverer 3.0 software to obtain matched peak data. The parameters were set as follows: quality range, 100–1,500; quality deviation, 5 × 10^–6^; retention time deviation, 0.05 min; SNR threshold, 3. Peak area normalization: Normalized data were imported into SIMCA-P 13.0 software for principal component analysis (PCA), partial least-squares discriminant analysis (PLS-DA), and orthogonal partial least squares discriminant analysis (OPLS-DA). OPLS-DA S-plot chart VIP >1 and independent-samples *t*-test *p* < 0.05 were used to screen differential metabolites. The HMDB database and the mass spectrometry ion fragments were used to identify the selected compounds, and SPASS 16.0 software was used to conduct one-way analysis of variance (ANOVA) for differential metabolite data. Pathway enrichment analysis was performed via the Metaboanalyst platform, and the related pathways of differential metabolites were analyzed by using the KEGG online database.

## Results and Discussion

### Determination of Polysaccharides in Total APS

The standard curve of Glc measured by the phenol–sulfuric acid method is showed in Supplementary Figure S1, the linear regression equation of Glc is *A* = 7.6031 C + 0.0337 (*R* ^2^ = 0.9963). The results show that the Glc solution has a good linear relationship in the concentration range of 0.01–0.1 mg mL^−1^. The calculated total polysaccharide content is 74.6%.

### Determination of Protein Content in Total APS

The protein standard curve is showed in Supplementary Figure S2, the linear regression equation of the obtained protein standard is *A* = 0.4397 C + 0.011 (*R* ^2^ = 0.9971). The calculated protein content of the total polysaccharide is 0.42%.

### Determination of the Molecular Weight Distribution of Total APS

**FIGURE 1:**
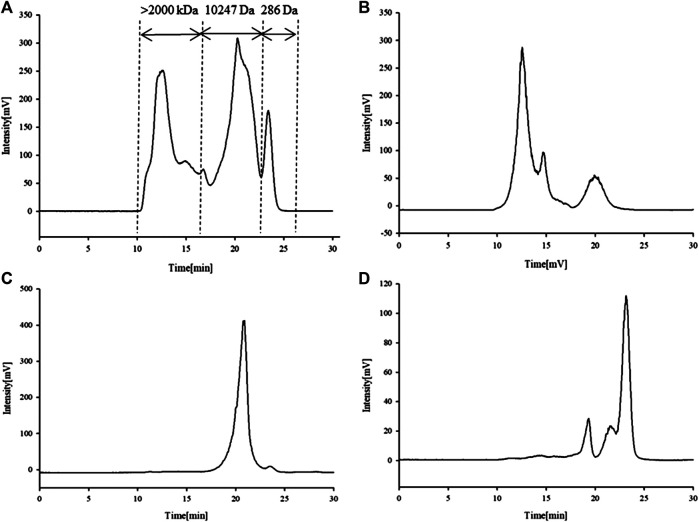
Chromatogram of APS determined high-performance liquid chromatography (HPLC-ELSD). APS **(A)**, APS-I **(B)**, APS-II **(C)**, APS-III **(D)**.

The standard curve of the dextran reference substance measured by HPGFC is showed in Supplementary Figure S3. The linear regression equation for the dextran reference substance is lgMw = −0.5336 *t*_*R*_ + 14.898 (*R* ^2^ = 0.9901). The calculated molecular weight distribution of total APS is divided into three parts (Figure 1A); here, the molecular weight of APS-I is >2,000 kDa, that of APS-II is 1.02 × 10^4^ Da, and that of APS-III is 286 Da.

### Purity and Molecular Weight of Different APS Components

Ultrafiltration yielded three APS fractions of different molecular weights: APS-I (molecular weight, >2000 kDa; purity, 85.2%), APS-II (molecular weight, 1.02 × 10^4^ Da; purity, 97%), and APS-III (molecular weight, 286 Da; purity, 84.6%). The content ratio of APS of different molecular weights is shown in Figures 1B–D specifically, APS-I:APS-II:APS-III ≈ 40:50:10%.

### Monosaccharide Composition of the Total Polysaccharide and APS of Different Molecular Weights

**FIGURE 2:**
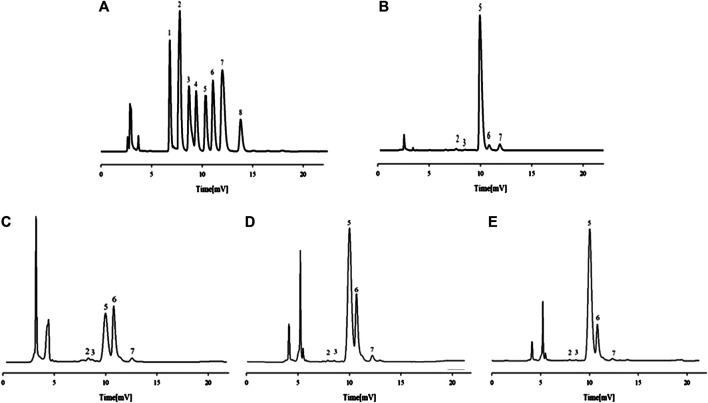
HPLC chromatograms of the monosaccharide control substance mixture **(A)** and monosaccharide composition of APS **(B)**, APS-I **(C)**, APS-II **(D)** and APS-III **(E)**. 1 - mannose, 2-rhamnose, 3-galacturonic acid, 4-N-acetylglucosamine, 5-glucose, 6-galactose, 7-arabinose, 8-fucose.

The chromatograms of eight standard monosaccharide mixtures (Figure 2A) and the monosaccharide characteristic map of total APS (Figure 2B) are determined by PMP pre-column derivatization−HPLC. The monosaccharide components of total APS include Rha, GalA, Glc, Gal, and Ara at a ratio of 0.43:0.23:19.36:0.69:1. The chromatograms of monosaccharide composition of APS of different molecular weights (Figures 2B–D) indicate that APS-I has a Rha:GalA:Gal:Glc:Ara ratio of 0.1:0.39:13.4:17.2:1, APS-II has a Rha:GalA:Gal:Glc:Ara ratio of 0.14:0.14:9.6:24.04:1, and APS-III has a Rha:GalA:Gal:Glc:Ara ratio of 0.375:0.375:18.8:90.5:1.

### Infrared Spectrum Analysis of APS of Different Molecular Weights

**FIGURE 3:**
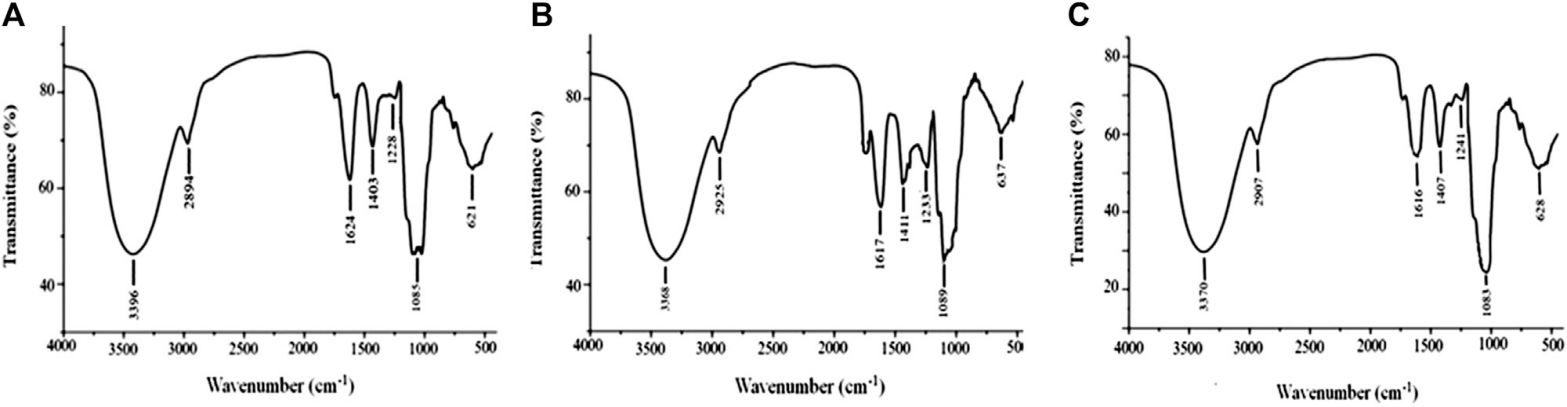
Infrared spectra of APS-I **(A)**, APS-II **(B)**, and APS-III **(C)**.

Stable FT-IR technology is widely used in chemical-bond and structural analysis. As shown in Figure 3A, APS-I has a strong absorption peak at 3,368 cm^−1^, which corresponds to O–H stretching vibration. The weak absorption at 2,936 cm^−1^ corresponds to C–H stretching vibration. The absorption peak at 1,629 cm^−1^ corresponds to the characteristic vibrations of the carboxyl group (COO–), which indicates the presence of uronic acid in APS. The peaks at 1,411 and 1,233 cm^−1^ correspond to the variable-angle vibrations of C–H and O–H, respectively, and the peak at 1,089 cm^−1^ represents the vibrations of C–O–H. The same peaks are observed in the spectra of APS-II (Figure 3B) and APS-III (Figure 3C). Therefore, APS-I, APS-II, and APS-III have similar functional groups.

### Methylation Analysis of APS of Different Molecular Weights

The mass spectra of the fragment ions of the three APS components analyzed by GC–MS methylation are compared with the mass spectra in the CCRC database (https://www.ccrc.uga.edu/specdb/ms/pmaa/pframe.html). Tables 2–4 respectively summarize the connection mode of monosaccharides of APS-I, APS-II, and APS-III.

**TABLE 2:** Analysis of APS-I methylation results.

No	*t*_R_/min	Sugar	Methylated sugar	Mass fragment m/z	Molar ratio	Linkages type
1	12.87	Gal-	2,6-Me_2_-Gal	74, 87, 118, 129, 143, 160, 185, 203, 305	5.66	→3,4)-d-Gal*p*-(1→
2	15.46	Ara-	2,3,5-Me_3_-Ara	71, 75, 87, 102, 118, 129, 135, 161	3.21	Ara*f*-(1→
3	19.91	Ara-	2,3-Me_2_-Ara	59, 71, 87, 102, 118, 129, 142, 162, 189	2.78	→5)-d-Ara*f*-(1→
4	24.87	Glc-	2,4,6-Me_3_-Glc	59, 87, 101, 118, 129, 157, 161, 234, 277	10.37	→3)-d-Glc*p*-(1→
5	26.43	Gal-	2,3,4-Me_3_-Gal	59, 71, 87, 102, 118, 129, 162, 189, 233	7.62	→6)-d-Gal*p*-(1→
6	27.25	Glc-	2,3,6-Me_3_-Glc	59, 71, 87, 99, 118, 129, 142, 173, 233	4.45	→4)-d-Glc*p*-(1→

**TABLE 3:** Analysis of APS-II methylation results.

No	*t*_R_/min	Sugar	Methylated sugar	Mass fragment m/z	Molar ratio	Linkages type
1	13.09	Gal-	2,6-Me_2_-Gal	74, 87, 118, 129, 143, 160, 185, 203, 305	2.36	→3,4)-d-Gal*p*-(1→
2	19.84	Ara-	2,3-Me_2_-Ara	59, 71, 87, 102, 118, 129, 142, 162, 189	3.08	→5)-d-Ara*f*-(1→
3	22.74	Glc-	2,3,4,6-Me_4_-Glc	59, 71, 87, 102, 118, 129, 145, 162, 205	5.77	D-Glc*p*-(1→
4	26.63	Gal-	2,3,4-Me_3_-Gal	59, 71, 87, 102, 118, 129, 162, 189, 233	4.42	→6)-d-Gal*p*-(1→
5	27.51	Glc-	2,3,6-Me_3_-Glc	59, 71, 87, 99, 118, 129, 142, 173, 233	14.56	→4)-d-Glc*p*-(1→
6	30.7	Glc-	2,3,4-Me_3_-Glc	59, 71, 87, 99, 118, 143, 159, 173, 189	6.23	→6)-d-Glc*p*-(1→

**TABLE 4:** Analysis of APS-Ⅲ methylation results.

No	*t*_R_/min	Sugar	Methylated sugar	Mass fragment m/z	Molar ratio	Linkages type
1	19.17	Ara-	2,3-Me_2_-Ara	59, 71, 87, 102, 118, 129, 142, 162, 189	1.07	→5)-d-Ara*f*-(1→
2	26.34	Gal-	2,3,4-Me_3_-Gal	59, 71, 87, 102, 118, 129, 162, 189, 233	4.58	→6)-d-Gal*p*-(1→
3	27.35	Glc-	2,3,6-Me_3_-Glc	59, 71, 87, 99, 118, 129, 142, 173, 233	26.94	→4)-d-Glc*p*-(1→

Methylation analysis reveals that the main chain of APS-I is →3)-d-Glc-(1→,→4)-d-Glc-(1→,→6)-d-Gal-(1→,→3,4)-d-Gal-(1→, and a small amount of arabinose. The main chain of APS-II is d-Glc*p*-(1→,→4)-d-Glc-(1 analysis revea and a small amount of arabinose and galactose. The main chain of APS-III is →4)-d-Glc-(1→.

The biological activity of polysaccharides is closely related to its structural characteristics (such as monosaccharide composition, molecular weight, connection mode, solubility, etc.) (He et al., 2020). Studies have shown that the presence of α-d-(1→4) and α-d-(1→6) glycosidic bonds in polysaccharides may be related to immunomodulatory activity. For example, the water-soluble polysaccharide extracted from sea clams, whose backbone is α-d-(1, 4)-glucan, can enhance the proliferation of spleen cells and increase the secretion of cytokines from lymphocytes, thereby enhancing immune activity (Wang et al., 2018). The main chain of polysaccharide extracted from cultured *Rhizopus nigricans* is α-d-(1, 4)-glucan, with a small amount of branching chains, and can improve the immune function of tumor-bearing mice and significantly inhibit the growth of transplanted tumors (Wei et al., 2018). The polysaccharides containing α-d-(1, 4)-glucan and α-d-(1, 6)-glucan in the main chain extracted from *maca* can act on the membrane receptors TLR2, CR3 and MR on phagocytes to enhance the phagocytosis (Zhang et al., 2016). The glucan with the main chain of α-d-(1, 4)-glucan and the branching chain α-d-(1, 6)-glucan isolated from the *Tinospora cordifolia* can activate different subsets of lymphocytes, such as natural killer cells, T lymphocytes and B lymphocytes (Nair et al., 2004). In summary, the main chain of these immunologically active polysaccharides is mainly composed of α-(1, 4)-glucan, so we speculate that the strong immunomodulatory activity of these polysaccharides may be related to the α-1, 4 glycosidic bonds in their structure.

The molecular weight and structural conformation of polysaccharides affect the solubility of polysaccharides and the viscosity of the solution, which in turn affects the absorption and biological activity of polysaccharides *in vivo* (Yu et al., 2018). High-viscosity solutions will hinder the diffusion and absorption of polysaccharides, low-molecular-weight polysaccharides have higher solubility than high-molecular-weight polysaccharides, and have higher solubility and lower viscosity in water, so they are more easily absorbed by the body when they function *in vivo*, and have a higher affinity with phagocytes, which helps immune stimulation (Mueller et al., 2000; Zhou et al., 2004).

In this study, APS-II with a low-molecular-weight (1.02 × 10^4^ Da) has a high solubility in water, which is beneficial to its immunomodulatory activity *in vivo*. Moreover, the branching degree of APS-II is lower than that of APS-I, and studies have shown that linear or branching α-d-(1, 4)-glucan and α-d-(1, 6)-glucan both show immunostimulatory activity, and the lower branching degree have higher immune enhancing activity, which is the structural feature of α-(1, 4)-glucan with higher immune-stimulating activity (Yan et al., 2011; Ferreira et al., 2015).

### Mice blood routine indicators

**FIGURE 4:**
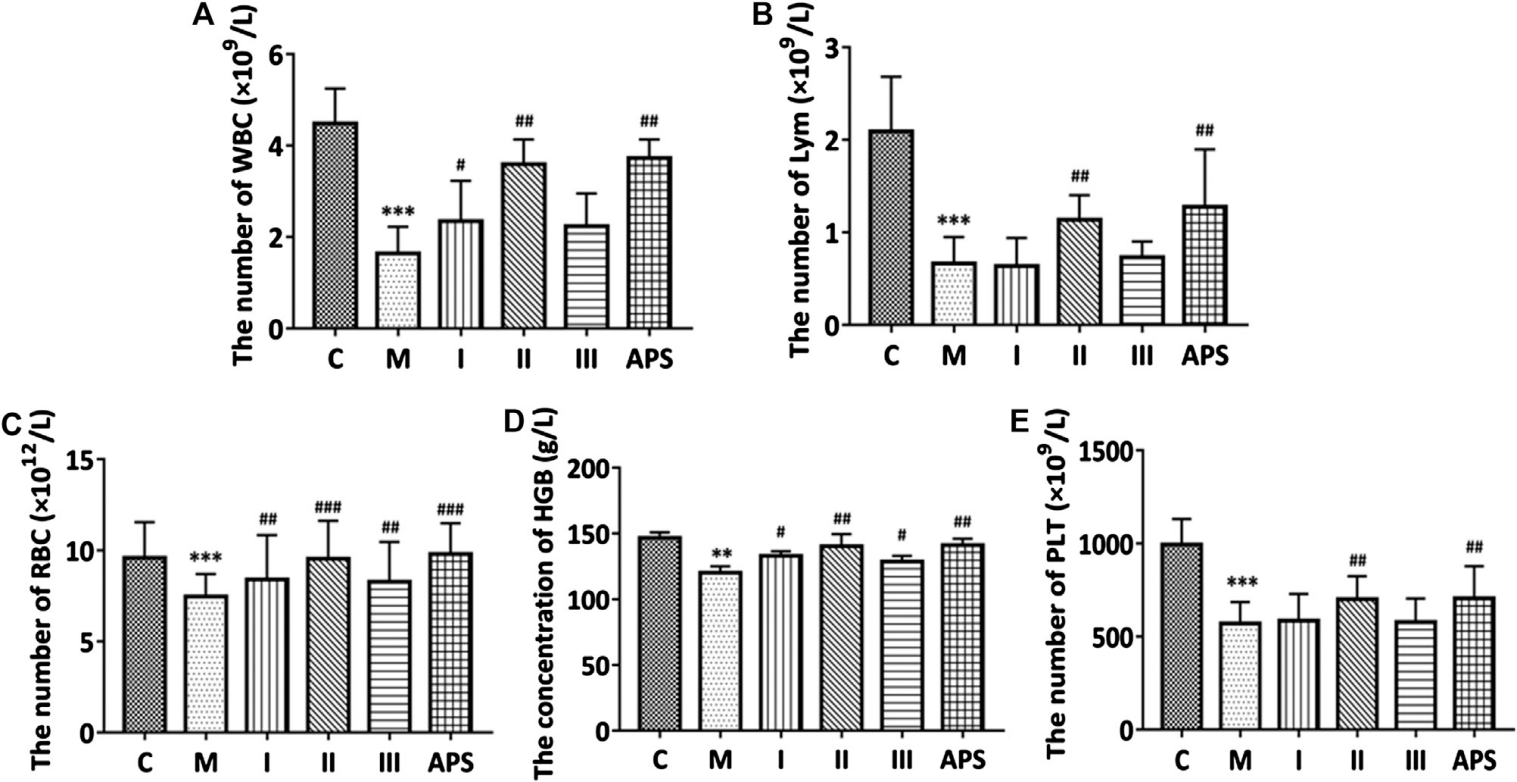
Comparison of the routine blood indices of each group. White blood cells **(A)**, lymphocytes **(B)**, red blood cells **(C)**, hemoglobin **(D)**, platelets **(E)**. **C**: control group, M: model group, I: APS-I, II: APS-II, III: APS-III. n = 6, ± *s*, *p < 0.05, **p < 0.01, ***p < 0.001 *vs*. the control group; ^#^ *p* < 0.05, ^##^ *p* < 0.01, ^###^ *p* < 0.001 vs. the model group.

The routine blood indices of the mice include white blood cells, lymphocytes, red blood cells, hemoglobin, and platelets. The data of each group are shown in Figure 4. The blood indices of the M group are significantly lower than those of the C group (*p* < 0.01), which indicates that the cyclophosphamide-induced immunosuppression model is effective. Compared with the M group, the drug-administration groups (APS with different molecular weights) could improve routine blood indicators to varying degree. Among the groups tested, APS-II shows the best effect, and its efficacy is similar to that of total APS. By contrast, APS-III shows the weakest effect.

### Mice body weight

**FIGURE 5:**
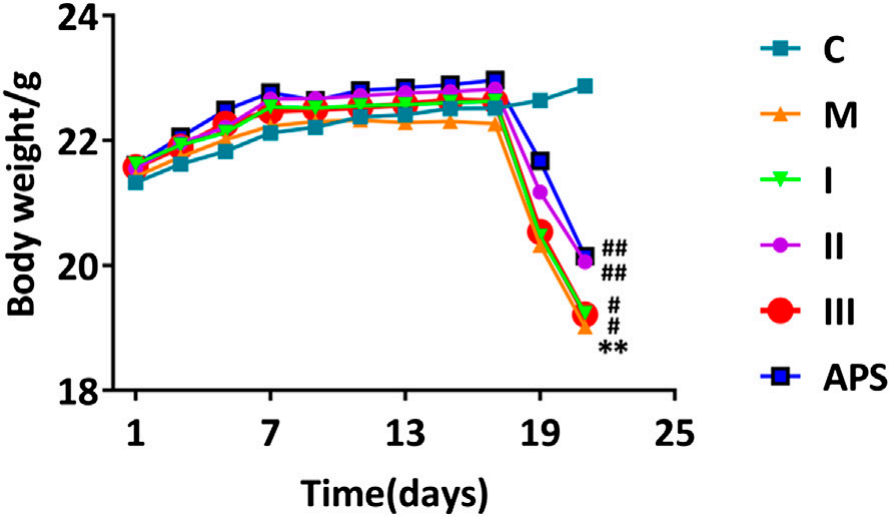
Effect of different APS components on mouse body weight. C: control group, M: model group, I: APS-I, II: APS-II, III: APS-III. n = 9, ± *s*, *p < 0.05, **p < 0.01, ***p < 0.001 vs. the control group; ^#^ *p* < 0.05, ^##^ *p* < 0.01, ^###^ *p* < 0.001 vs. the model group.

Changes in the body weight of mice in each group are shown in Figure 5. The weights of mice in the C group show an upward trend with time. After injection of cyclophosphamide, the weights of mice in the M group decline. The weights of mice in the drug-administrations groups also a decline, but the degree of decline observed in these groups is lower than that in the M group. Changes in the weight of mice treated with APS-II are similar to those treated with the total APS. Compared with these treatments, the adjustment effect of APS-I and APS-III is weaker.

### Mice Immune Organ Weight

**FIGURE 6:**
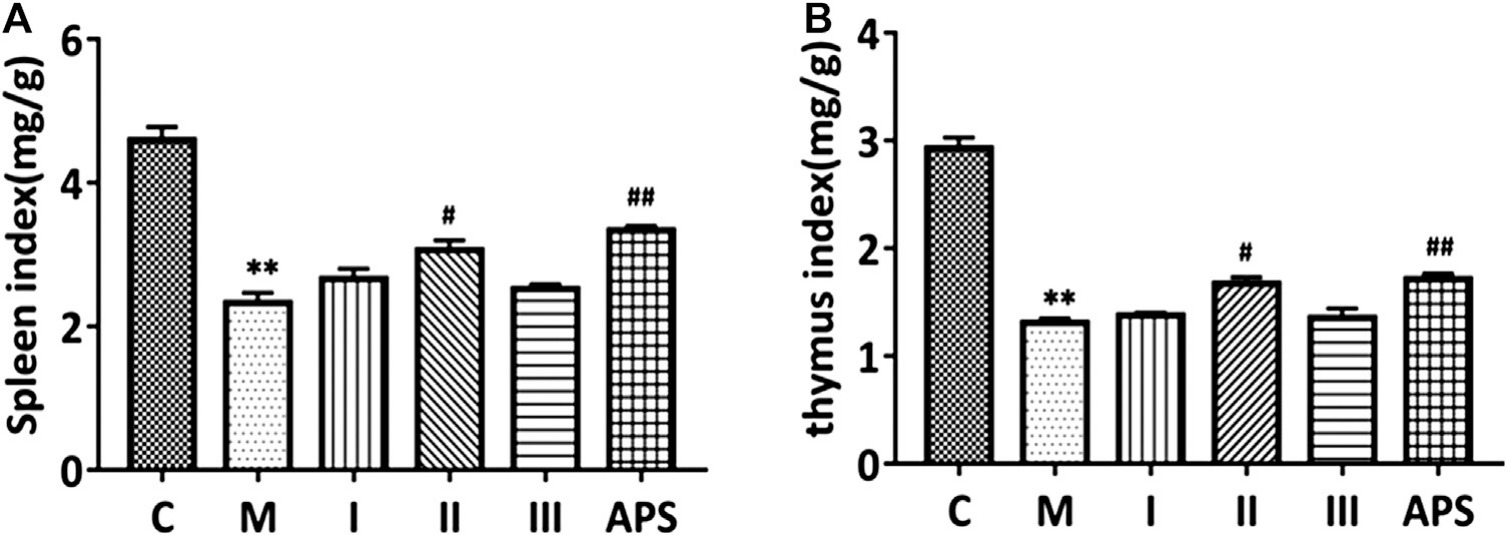
Comparison of spleen **(A)** and thymus **(B)** indices between groups. C: control group, M: model group, I: APS-I, II: APS-II, III: APS-III. *n* = 6, ± *s*, *p < 0.05, **p < 0.01, ***p < 0.001 vs. the control group; ^#^ *p* < 0.05, ^##^ *p* < 0.01, ^###^ *p* < 0.001 vs. the model group.

The spleen and thymus indices were calculate as spleen weight (mg)/mouse body weight (g) and thymus weight (mg)/mice body weight (g), respectively. As shown in Figure 6, the spleen and thymus indices of the M group are significantly lower than those of the C group (*p* < 0.01), which indicates that cyclophosphamide damages the immune organs of the model mice. After administration of APS of different molecular weights, the spleen and thymus indices of the mice show different degrees of recovery. Among the treatments, APS-II shows the best effect; indeed, its effect is similar to that APS (*p* < 0.05). This result indicates that APS-II can significantly improve the organ damage caused by cyclophosphamide.

### Stability testing of UHPLC Q-Exactive MS

Each batch of six test samples is randomly interspersed with a QC sample, and cluster analysis is performed on the QC samples to test the stability of the UHPLC Q-Exactive MS system. Ten ions are extracted from the six QC samples, and the RSDs of the retention times of these ions (t_R_), their mass-to-charge ratio (m/z), and relative peak areas are calculated to detect the stability of the LC-MS system. The results show that the QC samples are clustered together. The RSD of t_R_ is 0.27−1.14%, the RSD of m/z is 1.62 × 10^–7^−3.30 × 10^–7^, and the RSD of relative peak area is 3.11−25.06% (Table 5). These results confirm that the analytical method applied in this study has high repeatability and stability and is suitable for the analyses of large quantities of serum samples for metabolomics.

**TABLE 5:** Stability of the UHPLC-MS method determined using QC samples.

No.	*t*_R_/min	RSD (%)	RSD of relative peak areas/%	*m/z*	Ion	RSD (%)
1	1.28	1.1405	19.43	215.0398	[M−H]^−^	2.94 × 10^–5^
2	1.60	1.1037	25.06	132.0946	[M+H]^+^	3.09 × 10^–5^
3	6.63	0.3424	12.84	723.5102	[M−H]^−^	1.62 × 10^–5^
4	11.21	0.5977	11.86	274.2665	[M+H]^+^	1.88 × 10^–5^
5	16.74	0.3019	3.11	520.3325	[M+H]^+^	2.55 × 10^–5^
6	16.87	0.3404	9.27	588.3383	[M−H]^−^	1.78 × 10^–5^
7	17.17	0.3313	7.57	496.3324	[M+H]^+^	2.36 × 10^–5^
8	17.79	0.2863	8.00	540.3384	[M−H]^−^	2.16 × 10^–5^
9	18.75	0.2759	6.87	522.3482	[M+H]^+^	3.30 × 10^–5^
10	21.35	0.3165	5.54	524.3639	[M+H]^+^	2.23 × 10^–5^

### Statistical Analysis of Metabolic Data by PCA, PLS-DA and OPLS-DA

**FIGURE 7:**
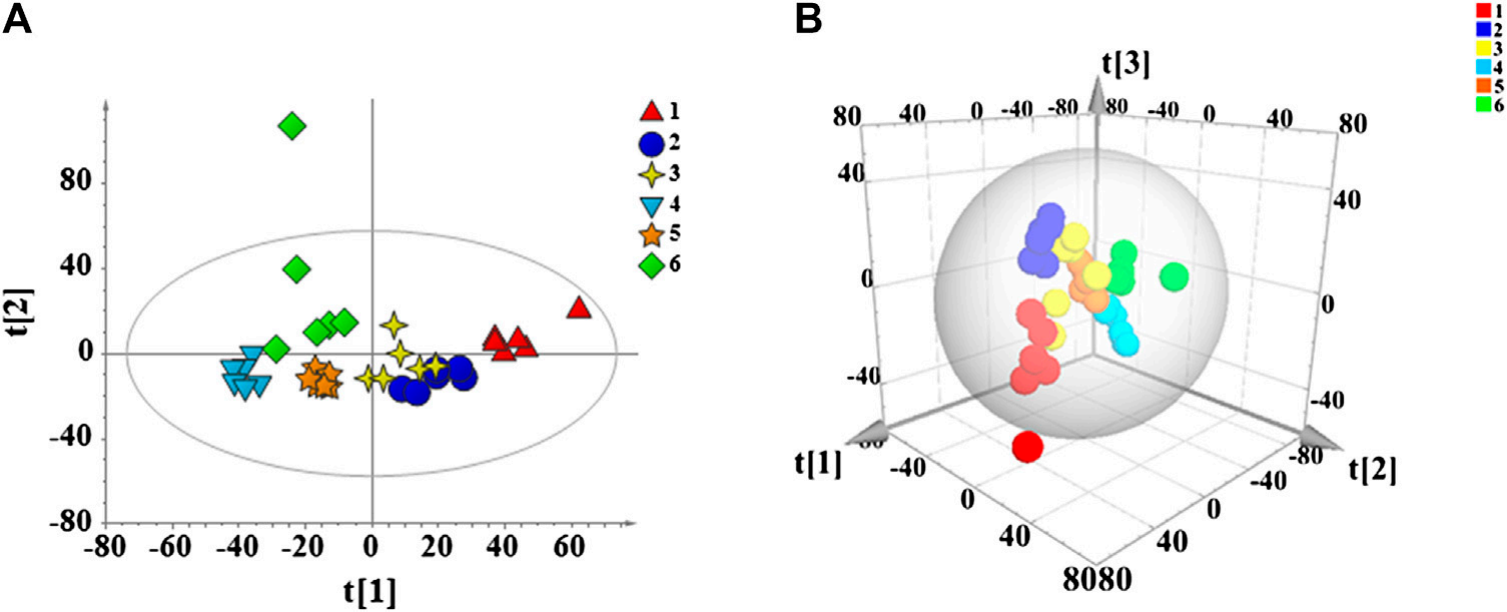
PCA score plots of all mouse serum samples. 2D diagram **(A)** and 3D diagram **(B)**. 1: Model group, 2: Control group, 3: APS, 4: APS-I, 5: APS-II, 6: APS-III.

Data are collected from each group of mice serum samples by UHPLC-Q Exactive Orbitrap MS to obtain the metabolic profile of each group. PCA is performed on all sample data to show their original classification status. As shown in Figure 7, the blank and model groups could be clearly separated, which means the cyclophosphamide-induced immunosuppression mouse model is successfully constructed. The drug administration group is also clearly separated from the model group. The 2D and 3D graph obtained by PCA clustering reveal that the APS-II and APS groups are closer to the blank control group after administration than other groups, thus suggesting that these groups have a certain callback effect on the mouse immunosuppression model.

**FIGURE 8:**
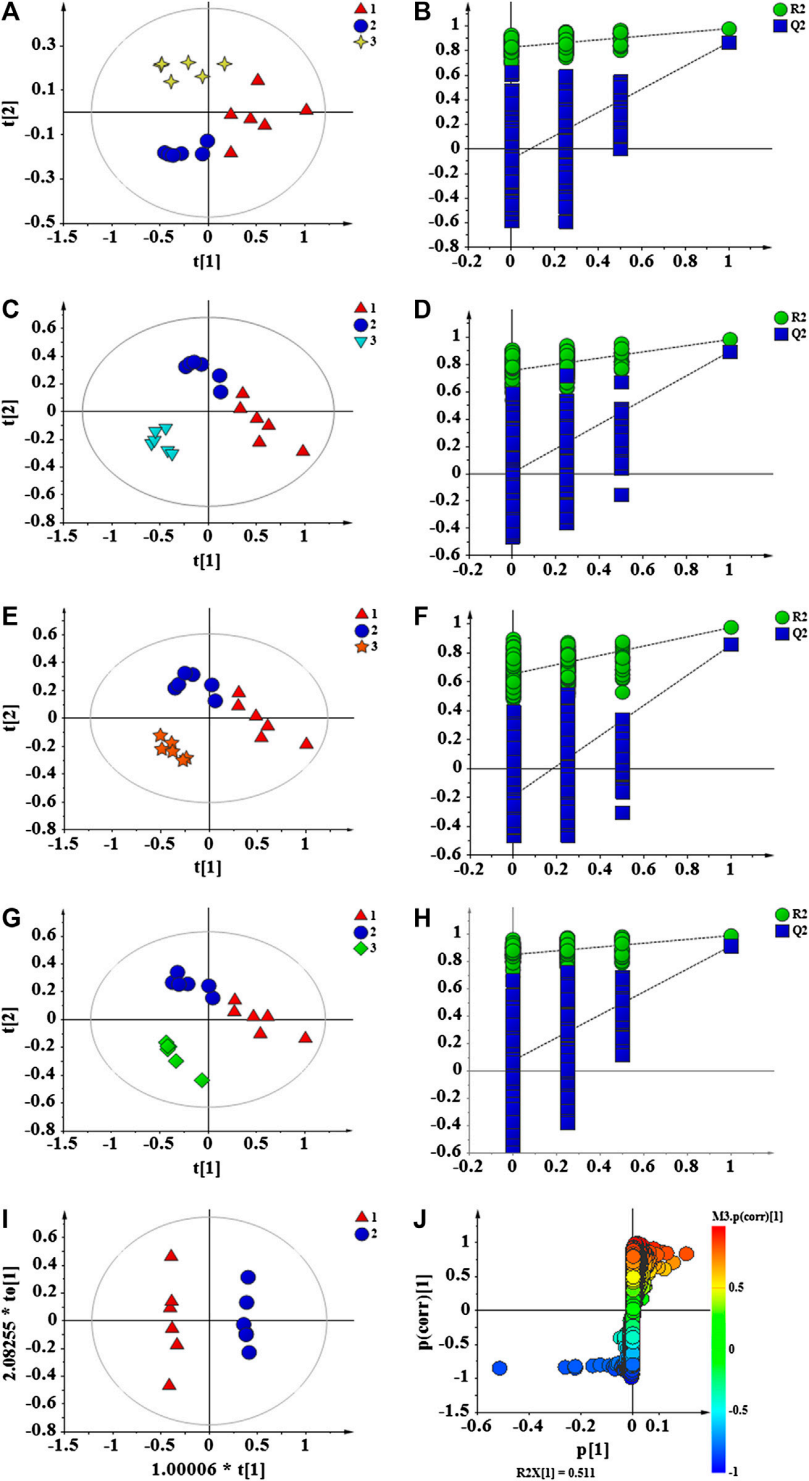
PLS-DA score plots of C, M and APS **(A)**; permutation test of C, M and APS **(B)**; PLS-DA score plots of C, M and APS-I **(C)**; permutation test of C, M and APS-I **(D)**; PLS-DA score plots of C, M and APS-II **(E)**; permutation test of C, M and APS-II **(F)**; PLS-DA score plots of C, M and APS-III **(G)**; permutation test of C, M and APS-III **(H)**; OPLS-DA score plots of C and M **(**I**)**; S-plot of C and M **(J)**. C: Control group, M: Model group. 1 (Triangle) = M, 2 (circle) = C, 3 (4-plot star) = APS, 3 (inverted triangle) = APS-I, 3 (5-plot star) = APS-II, 3(diamond) = APS-III.

The data of each group were analyzed by PLS-DA to achieve accurate cluster discrimination between groups. As shown in Figures 8A,C,E,G, the control group (C), model group (M), and drug administration group could be clearly separated. This result confirms that the model is effective and reliable. Next, 200 random arrangement experiments were performed on the model data. Figures 8B,D,F,H reveal that the *R* ^2^ and *Q* ^2^ on the left side of the random variable Y are lower than the original value on the right side and that the slope of the model is slightly large. This finding indicates that the model is not over-fitted and that it is effective and reliable. OPLS-DA analysis was performed on the blank and model groups to determine metabolic variables related to the immune insult model. The results showed that the C and M groups are clearly separated (Figure 8I), the VIP >1 in S-plot (Figure 8J) combined with independent-samples *t*-test *p* < 0.05 of blank group and model group to find related differential metabolites.

### Identification and Analysis of 29 Differential Metabolites

**FIGURE 9:**
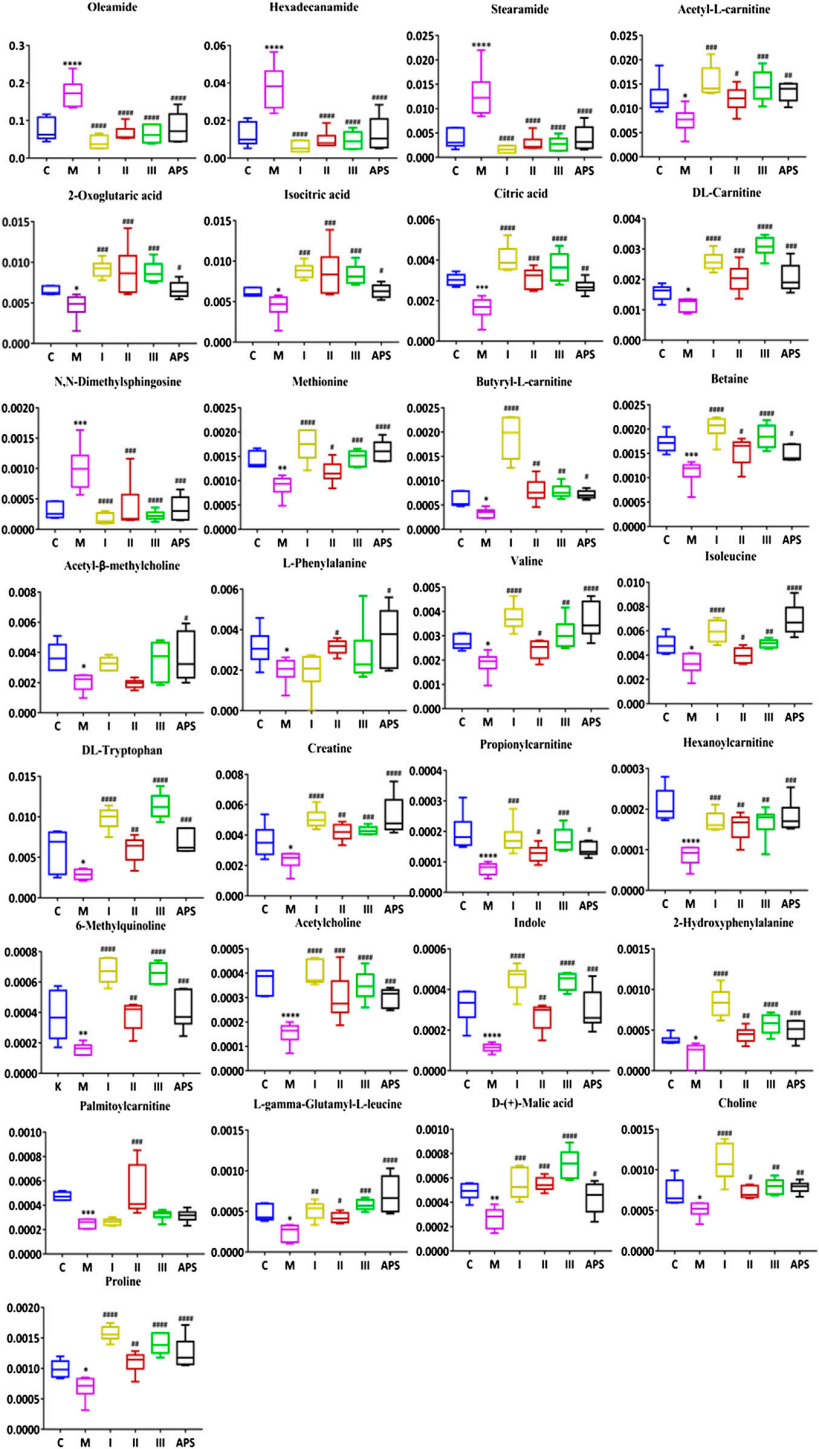
Comparison of the normalized peak areas of differential metabolites in each group. C: control group, M: model group, I: APS-I, II: APS-II, III: APS-III. *n* = 6, ± *s*, *p < 0.05, **p < 0.01, ***p < 0.001 vs. the control group; ^#^ *p* < 0.05, ^##^ *p* < 0.01, ^###^ *p* < 0.001 vs. the model group.

According to VIP >1 combined with independent-samples *t*-test *p* < 0.05 of blank group and model group, and identified by the HMDB database and metabolite secondary fragment ions, a total of 29 differential metabolites related to the cyclophosphamide-induced immune insult model are identified (Table 6). One-way ANOVA is performed on these metabolites. As shown in Figure 9, compared with the C group, the levels of oleamide, hexadecanamide, stearamide, *N*,*N*-dimethylsphingosine increased in the M group. Significant callback is observed after APS of different molecular weights administration. Compared with the C group, acetyl-l-carnitine, 2-oxoglutaric acid, isocitric acid, DL-tryptophan, isoleucine, creatine, acetyl-β-methylcholine, l-phenylalanine, valine, citric acid, DL-carnitine, betaine, butyryl-l-carnitine, methionine, palmitoylcarnitine, proline, choline, l-γ-glutamyl-l-leucine, D-(+)-malic acid, 2-hydroxyphenylalanine, 6-methylquinoline, indole, acetylcholine, propionylcarnitine, and hexanoylcarnitine levels are reduced in the M group. Significant callback is observed after APS of different molecular weights administration. Among them, treatment with APS resulted in the callback of 24 metabolites to normal levels, APS-I has a total of 13 callbacks, APS-II has a total of 25 callbacks, APS-III has 19 callbacks. The above results show that APS of different molecular weights produce a certain degree of immune repair activity when applied at the same dose. Among the samples tested, APS-II shows the best effect, total polysaccharides APS has the second best effect.

**TABLE 6:** Differential metabolites detected in mouse serum by UHPLC-Q-Orbitrap-MS/MS.

No.	Metabolite	*t*_R_/min	Formula	*m/z*	Ion	VIP (>1)
1	Oleamide	26.098	C_18_H_35_NO	282.2717	[M+H]^+^	30.4936
2	Hexadecanamide	25.11	C_16_H_33_NO	256.25601	[M+H]^+^	15.4175
3	Stearamide	28.837	C_18_H_37_NO	284.28736	[M+H]^+^	9.10991
4	Acetyl-l-carnitine	1.597	C_9_H_17_NO_4_	204.11568	[M+H]^+^	5.74342
5	2-Oxoglutaric acid	1.6	C_5_H_6_O_5_	145.02105	[M−H]^−^	3.69693
6	Isocitric acid	1.582	C_6_H_8_O_7_	191.02656	[M−H]^−^	3.43281
7	dl-Tryptophan	5.783	C_11_H_12_N_2_O_2_	205.08979	[M+H]^+^	4.79669
8	Isoleucine	1.604	C_6_H_13_NO_2_	132.09464	[M+H]^+^	3.60728
9	Creatine	1.351	C_4_H_9_N_3_O_2_	132.06951	[M+H]^+^	2.98949
10	Acetyl-β-methylcholine	1.576	C_8_H_17_NO_2_	160.12585	[M+H]^+^	3.80388
11	l-Phenylalanine	1.656	C_9_H_11_NO_2_	166.07894	[M+H]^+^	2.81315
12	Valine	1.591	C_5_H_11_NO_2_	116.07912	[M−H]^−^	2.72165
13	Citric acid	2.349	C_6_H_8_O_7_	191.02668	[M−H]^−^	3.69294
14	dl-Carnitine	1.313	C_7_H_15_NO_3_	162.10514	[M+H]^+^	1.71998
15	*N*,*N*-Dimethylsphingosine	29.381	C_20_H_41_NO_2_	328.50292	[M+H]^+^	2.51312
16	Betaine	1.287	C_5_H_11_NO_2_	118.07911	[M+H]^+^	2.34196
17	Butyryl-l-carnitine	5.743	C_11_H_21_NO_4_	232.14691	[M+H]^+^	1.48593
18	Methionine	1.602	C_5_H_11_NO_2_S	150.051	[M+H]^+^	2.19918
19	Palmitoylcarnitine	18.383	C_23_H_45_NO_4_	400.33466	[M+H]^+^	1.54599
20	Proline	1.358	C_5_H_9_NO_2_	116.0635	[M+H]^+^	1.5666
21	Choline	1.259	C_5_H_13_NO	104.09999	[M+H]^+^	1.24409
22	l-γ-Glutamyl-l-leucine	5.773	C_11_H_20_N_2_O_5_	259.13695	[M−H]^−^	1.42737
23	d-(+)-Malic acid	1.56	C_4_H_6_O_5_	133.02075	[M−H]^−^	1.4384
24	2-Hydroxyphenylalanine	3.135	C_9_H_11_NO_3_	182.07381	[M+H]^+^	1.16375
25	6-Methylquinoline	5.789	C_10_H_9_N	144.07345	[M+H]^+^	1.31423
26	Indole	5.777	C_8_H_7_N	118.05792	[M+H]^+^	1.42674
27	Acetylcholine	1.38	C_7_H_15_NO_2_	146.11026	[M+H]^+^	1.48906
28	Propionylcarnitine	1.619	C_10_H_19_NO_4_	218.13125	[M+H]^+^	1.05437
29	Hexanoylcarnitine	6.526	C_13_H_25_NO_4_	260.17825	[M+H]^+^	1.11086

### Correlation and Metabolic Pathway Analyses of Differential Metabolites

**FIGURE 10:**
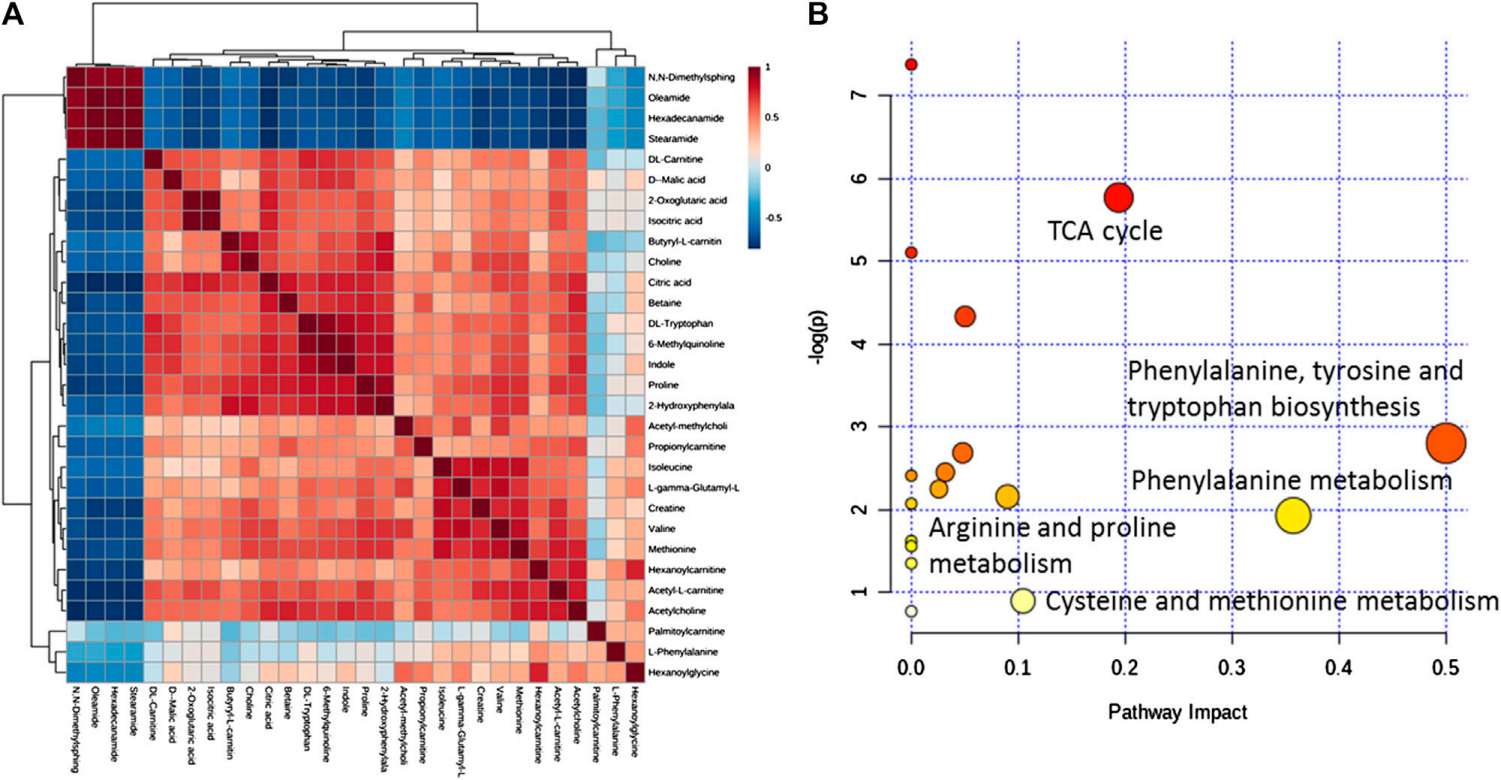
Correlation analysis of differential metabolites in mouse serum **(A)**. MetPA analysis of metabolic pathways **(B)**.

Metaboanalyst is used to analyze correlations among differential metabolites in mouse serum. In the heat map of Figure 10A, a darker color indicates a stronger correlation between two metabolites, and a lighter color indicates a weaker correlation. The brown block indicates the content of a certain metabolite increases, and the content of the metabolite with a strong correlation will increase as a positive correlation, and the blue block indicates the content of the metabolite with a strong correlation will decrease as a negative correlation. In this figure, differential metabolites such as *N*,*N*-dimethyl sphingosine, oleamide, hexadecamide, and stearamide have a strong positive correlation with each other and a strong negative correlation with the 25 other metabolites. A strong positive correlation between 2-oxoglutaric acid and isocitrate, a strong positive correlation between 6-methylquinoline and DL-carnitine, and a strong positive correlation between hexanoyl carnitine and cetylamide, among others, are also noted. The correlations between these differential metabolites indicate that the immune-repairing activity of APS is related to multiple metabolic pathways that are interrelated and interact with each other to produce combined immune enhancement effects. Twenty-nine differential metabolites in mouse serum are analyzed by pathway enrichment, as shown in Figure 10B, the impact value of metabolic pathway (impact value) >0.1, which is regarded as the main metabolic pathway related to immune regulation. In this analysis, the darker the dot color, the greater the number of metabolites involved in this pathway; in addition, the larger the dot, the more obvious the effect of this pathway (Xia and Wishart, 2010). A total of 17 differential metabolite-related pathways are found, and five major metabolic pathways are determined by integrating Holm *p* values, false discovery rates, and impact values. More metabolites participate in the TCA cycle, phenylalanine, tyrosine and tryptophan biosynthetic, and phenylalanine metabolic pathways, and the role of these pathways is obvious; thus, they are main metabolic pathways. The arginine + proline and cysteine + methionine metabolic pathways also have an influence value >0.1, which means these pathways are important metabolic pathway related to immune regulation by APS.

Effect of APS on the immune function through the phenylalanine, tyrosine and tryptophan biosynthetic, and phenylalanine metabolic pathways

In this study, the level of phenylalanine in the serum of mice was reduced after cyclophosphamide modeling and returned to normal levels after the administration of APS-II and APS. Phenylalanine, one of the essential amino acids in the body, is mostly converted into tyrosine by the action of phenylalanine hydroxylase (PAH). It synthesizes important neurotransmitters and hormones, together with tyrosine, and participates in the body’s sugar and fat metabolism (Li and Li, 2006). Related studies have shown that the content of phenylalanine in serum is closely related to the degree of immune activation. During the induction of specific and non-specific immune responses, the interferon IFN-γ activating enzyme GCH1 generates neoopterin in activated immune cells, while 5,6,7,8-tetrahydrobiopterin (BH_4_) is produced in most cells. BH_4_, a cofactor of PAH, tyrosine hydroxylase, and tryptophan hydroxyl, is extremely sensitive to oxidation. Oxidative stress is usually caused by immune activation. Activated immune cells release a large amount of reactive oxygen species and oxidize BH_4_ in normal cells, thereby inhibiting the activity of PAH. The conversion of phenylalanine to tyrosine is then inhibited, and the content of phenylalanine increases (Zangerle et al., 2010; Strasser et al., 2016). APS can promote the maturation of dendritic cells (DC). After DC maturation, upregulation of co-stimulatory molecules, such as CD80, CD40, and CD86, which are necessary for T cell activation (Luo and Shuo, 2009). APS administration may increase the production and release of neopterin and BH_4_ by repairing the stress function of mouse immune cells, thereby regulating the phenylalanine metabolic pathway and returning phenylalanine contents to normal levels.

### APS Participates in Immune Regulation Through Cysteine and Methionine Metabolic Pathways

Methionine, another essential amino acid for the human body, participates in protein synthesis and metabolism, immune regulation, and antioxidant functions in the body. After methionine enters the human body, it reacts with ATP under the catalysis of methionine adenosine transferase (MAT) to produce S-adenosylmethionine (SAM). SAM has an active methyl group that can participate in the synthesis and degradation of various hormones and neurotransmitters through various transmethylation functions; it also exerts anti-inflammatory and anti-oxidation functions. SAM is demethylated to generate S-adenosyl homocysteine (SAH), which removes adenosine to produce homocysteine (Hcy), some of which accepts methyl to regenerate methionine and form the methionine cycle. Another part of Hcy metabolizes to produce glutathione (GSH) and taurine (Peng et al., 2017). GSH can promote the production of IL-12 through antigen-presenting cells and enhance the function of T lymphocytes (Peterson et al., 1998), while taurine can change the balance between Th1 and Th2 cytokines and maintain the balance between the inflammatory and specific immune responses. Related studies have shown that Hcy, a marker of the Th-1 type immune response, is significantly positively correlated with serum neopterin (Grimble, 2006). After cyclophosphamide-induced immune insult, the immune function of mice decreased, and the methionine content in the serum of the mice decreased. The activity of the immune cells of the mice repaired after APS administration. The neopterin produced increases and positive feedback regulates Hcy, thereby indirectly recalling the methionine content to normal levels in the methionine cycle.

### APS Participates in Immune Regulation Through Arginine and Proline Metabolism

The metabolism of arginine in mammals is fairly complex. Arginine and its metabolites are the center of various metabolic pathways in the body. Studies have shown that the main metabolites of arginine are protein, urea, ornithine, citrulline, NO, creatine, glutamic acid, proline, polyamine, glutamic acid, agmatine, and homoarginine (Morris, 2016). Arginine internally synthesizes creatine in the body with glycine and methionine. Creatine is one of the most important substances in energy metabolism, arginine is hydrolyzed to ornithine and urea under the action of arginase, and ornithine acid can produce proline under the action of ornithine transaminase and then glutamic acid. Proline plays an important role in protein structure synthesis, metabolism, wound healing, antioxidant response, and the immune response (Wu G. et al., 2011; Rodríguez-Gómez et al., 2016). In this study, the levels of creatine and proline in the serum of mice after modeling are significantly lower than those of normal mice, and the arginine metabolic pathway is apparently disturbed. This effect may explain, at least in part, the decline in the immune ability of mice. Regulating the arginine metabolic pathway could restore creatine and proline levels to normal levels, and restore the immune ability of the mice.

### APS Promotes Immune Function Callback in Mice Through the Citric Acid Cycle

The citric acid cycle, also known as the TCA cycle, is a cyclic reaction system composed of a series of enzymatic reactions (Williams and Oneill, 2018). Macrophages and DCs can quickly switch from a resting state to an activated state when they play an immunomodulatory function, and the sign of immune cell activation is a change in intracellular metabolic pathways. TLR receptors on the surface of macrophages and DCs are activated during activation of macrophages and DCs, erobic glycolysis increases (Arts et al., 2016), and the intracellular glycolysis and pentose phosphate pathways are up-regulated; these changes affect two links in the TCA cycle. Moreover, the fatty acid oxidation and oxidative phosphorylation pathways are downregulated, which results in increased levels of citric acid, isocitrate, and ketoglutarate. In this study, the model mice have decreased immune function, show decreased immune cell activity, and are insensitive to immune-related metabolic pathways, after APS administration, normal function of immune cells is restored, and citric acid, isocitrate, and ketoglutarate contents return to normal levels.

## Conclusion

In this study, APS, APS-I, APS-II, and APS-III were prepared by water extraction, alcohol precipitation, and ultrafiltration separation and their molecular weight distribution, monosaccharide composition, and sugar chain attachment site were determined. A cyclophosphamide-induced immunosuppression model was used to investigate the mechanism of the immunomodulatory activity of APS of different molecular weights with reference to normal mice. Studies have shown that the main chains of APS-I, APS-II, and APS-III are →3)-d-Glc-(1→,→4)-d-Glc-(1→,→6)-d-Gal-(1→,→3,4)-d-Gal-(1→, d-Glc*p*-(1→,→(1lces have shown that the and →4)-D-Glc-(1→, respectively. Changes in blood routine indices, body weight, and the weight of immune organs before and after drug administration indicate that the three polysaccharides could enhance the immunity of mice to varying degrees. Among the three polysaccharides, APS-II, the molecular weight of which is approximately 10,000 Da, is the main active ingredient of APS, and its efficacy is similar to that of the total polysaccharide. APS-II shows stronger immunomodulatory activity than APS-I and APS-III, which may be related to its molecular weight, branching degree and α-1, 4 and α-1, 6 glycosidic bonds in its structure, according to polysaccharide receptor theory (Cao et al., 2019), APS-II with a low-molecular-weight and fewer branches is more soluble in water and absorbed by the body, so it is easier to combine with immune cell membrane receptors to exert stronger immune activity.

LC-MS technology was used to analyze the metabolic profile of the serum of each group of mice and screen differential metabolites and metabolic pathways related to immunosuppression. The results showed that five metabolic pathways and 29 differential metabolites are related to cyclophosphamide-induced immunosuppression. APS-II has similar regulatory effects with total APS and plays a major role in the immunomodulatory activity of the total APS. Analysis of differential metabolites and their metabolic pathways indicate that APS may mainly exert its immune activity in the body by directly affecting non-specific immunity. In the body, APS may regulate the activity of phagocytes and DCs and indirectly affect the specific immunity of the body through related metabolites. The polysaccharide shows strong overall immune function repair activity.

This article preliminarily determined the mechanism of action of APS. Several limitations must be considered when interpreting the results. First, the mechanism of action of the total polysaccharide must be thoroughly investigated considering transcriptomics and proteomics. Moreover, because of the complex structure of APS, its pharmacological process *in vivo* remains unclear and requires further study.

## Data Availability Statement

The original contributions presented in the study are included in the article/Supplementary Material, further inquiries can be directed to the corresponding authors.

## Author Contributions

KL and L-JC contributed equally to this work. KL and L-JC provided the concept and designed the study. L-JC, Y-XC, S-YL, and L-XS conducted the analyses. KL and L-JC wrote the manuscript. KL, L-JC, Y-XC, S-YL, and L-XS participated in data analysis. X-MQ and Y-GD provided oversight. KL, L-JC, Y-GD, and X-MQ revised and proofreading the manuscript. All authors have read and approve of the final manuscript.

## Funding

This study was financially supported by the National Natural Science Foundation of China (Grant No. 81872962), the National Key R&D Program of China (No. 2019YFC1710800), the China Postdoctoral Science Foundation Project (No. 2019M650851), the Key Projects of Key Research and Development Plan in Shanxi (No. 201603D311101), the Shanxi Province Technology Innovation Project of Excellent Talent (No. 201605D211030).

## Conflict of Interest

The authors declare that this research was conducted in the absence of any commercial or financial relationships that could be construed as a potential conflict of interest.
