# Supplementary material for: UHPLC Q-Exactive MS-Based Serum Metabolomics to Explore the Effect Mechanisms of Immunological Activity of Astragalus Polysaccharides With Different Molecular Weights
Source: Front Pharmacol. 2020 Dec 15;11:595692. doi: 10.3389/fphar.2020.595692 (PMC7774101; doi:10.3389/fphar.2020.595692)
Supplement: Supplementary file 1 [file datasheet1.docx]

**Figure S1** Standard of glucose of different concentrations.

**Figure S2** Standard curve of protein of different concentrations.

**Figure S3** Standard curve of the logarithm of the molecular weight of the dextran standard control and its corresponding retention time.
